# Predictors of Tobacco Use Behaviors Among Syrian Americans

**DOI:** 10.1177/1179173X261423430

**Published:** 2026-06-03

**Authors:** Isabel Nakoud, Cyrene Arputhasamy, Catherine M. Crespi, Jovana F. Mahho, Mellissa Withers, Burton O. Cowgill

**Affiliations:** 1 UCSD School of Medicine, 315531 University of California, San Diego, La Jolla, CA, USA; 2 Fielding School of Public Health, 25808 University of California, Los Angeles, Los Angeles, CA, USA; 3 UCI School of Medicine, 12219 University of California, Irvine, Irvine, CA, USA; 4 Keck School of Medicine, 12223 University of Southern California, Los Angeles, CA, USA

**Keywords:** tobacco use, cessation, hookah smoking, cigarettes, Syrian Americans

## Abstract

**Background:**

This study examines the prevalence and predictors of cigarette and hookah smoking among Syrian Americans, a growing U.S. immigrant population with historically high tobacco use.

**Objectives:**

To assess tobacco use behaviors and identify demographic and behavioral predictors of cigarette and hookah use, as well as key motivators for tobacco use, among Syrian American adults.

**Design:**

A cross-sectional survey study of Syrian American adults in 2 U.S. states.

**Methods:**

Data were collected from 919 Syrian American adults in Southern California and Florida between 2018 and 2019. Multinomial regression analyses were used to identify demographic and behavioral predictors of cigarette and hookah use.

**Results:**

Among participants, 16% were current cigarette users, and 37% were current hookah users, both exceeding U.S. national averages. Social occasions and flavored tobacco were key motivators for hookah use, with most participants perceiving hookah as equally or more harmful than cigarettes. Male gender, older age, longer U.S. residency among females, alcohol use, and lower education levels predicted cigarette smoking. Hookah use was associated with younger age, alcohol consumption, and the perception that hookah is less harmful than cigarettes, while health insurance and higher education were protective factors. Cigarette users were less likely to use hookah, whereas former cigarette users had a higher likelihood of hookah use. Education was a key factor in quitting both cigarettes and hookah. Individuals with health insurance were more likely to quit hookah, while those who perceived hookah as less harmful were less likely to quit.

**Conclusion:**

These findings emphasize the need for culturally tailored tobacco prevention and cessation programs for Syrian Americans, addressing misperceptions of hookah harms and leveraging community-specific motivators to develop effective interventions.

## Introduction

Cigarette smoking remains a leading cause of preventable death in the United States (U.S.).^1^ While trends in cigarette smoking are influenced by factors such as sex, race, ethnicity, and nativity, there is a notable lack of research on how these trends impact Syrian Americans, the third-largest group of Arab immigrants in the U.S.^2-4^

Since 2012, Syrian men have displayed the highest cigarette smoking rate (62%) among all their counterparts in Middle Eastern and North African countries.^5-7^ Syrian Americans may continue smoking at high rates in the U.S.; studies have noted both a higher smoking prevalence and lower cessation rate among Arab Americans compared to U.S. averages.^8,9^ A recent study on acculturation and smoking habits among immigrants in the U.S. found that the region of birth has a more substantial influence on smoking differences than the duration of residence in the U.S.^3^ Furthermore, the impact of sex on smoking outcomes is heightened within immigrant communities. For individuals born in the U.S., the likelihood of smoking is only marginally higher for males compared to females, yet among foreign-born groups, males are up to 6 times more likely to smoke.^3^ Similarly, the 2018 National Health Institute Survey demonstrated that Arab American males exhibited the highest national rate at 26%, while Arab American females had the lowest prevalence at 8%.^10^

Beyond cigarette smoking, other forms of tobacco use, such as hookah (also known as narghile or water-pipe smoking), have also gained popularity in the U.S. This may be due to the perception that hookah is less harmful, less addictive, and more socially acceptable than cigarette smoking.^11-15^ Prior studies also suggest that cigarette smoking and alternative tobacco use (cigars, e-cigarettes, etc.) may increase the likelihood of hookah smoking.^14-22^

The rise of hookah smoking, globally and in the U.S., poses additional health concerns. Despite being perceived as less harmful than cigarettes, hookah smoking is associated with similar adverse health effects.^23,24^ In the U.S., Arab Americans exhibit 2-3 times higher hookah smoking prevalence than the general U.S. population.^25,26^ Across the globe, Syrians, historically avid users of hookah, are among the top hookah users.^7,25,27,28^

While studies have investigated tobacco trends in the Arab world, research on Arab Americans in the U.S., particularly Syrian Americans, is limited.^26,29^ Given the high smoking rates, Syrian Americans may be at elevated risk for smoking-related illnesses. Consequently, this study seeks to illuminate these disparities by examining cigarette and hookah smoking predictors within the Syrian American community in the U.S.

## Methods

### Participants and Data Collection

This study presents a secondary analysis of an existing, anonymous dataset. The data were originally collected between January 2018 and May 2019 using self-administered electronic and paper surveys distributed in English and Arabic to Syrian Americans at social events in cities across Southern California and Jacksonville, Florida. Inclusion criteria included age 18 or older, living in the U.S., and both parents of Syrian descent. A purposive convenience sampling approach was used to recruit participants, given the dispersed nature of the community and the absence of a comprehensive sampling frame. Participants were recruited at culturally relevant community events (eg, picnics, cultural gatherings, faith-based activities) that draw broad attendance from Syrian Americans across the region. No predetermined sample size was set prior to data collection. Consequently, the final sample size reflects the number of individuals who were recruited during the recruitment window.

The original data collection was approved by the University of Southern California Health Sciences Institutional Review Board. Participants completed anonymous surveys and provided implied informed consent by voluntarily completing the questionnaire after receiving an information sheet describing the study. For this manuscript, the secondary analysis of the existing anonymous dataset was reviewed by the Institutional Review Board at the University of California, Los Angeles and determined to be exempt under federal criteria for secondary research.

### Measures

The full survey instrument is provided in the Supplemental Materials. Data collected included demographics, immigration history, health insurance coverage, and use of alcohol, cigarettes, and hookah. For cigarette, and hookah use, data included frequency, age of initiation, and age of cessation if use was discontinued. Smoking statuses for hookah and cigarettes were categorized as current user, former user, or never user. These self-reported smoking status categories are commonly used in epidemiologic and community-based tobacco research, including studies of immigrant and Arab American populations.^9^ Cigarette use status was assessed using the question, ‘Do you smoke cigarettes?’ Responses were categorized as follows: individuals who answered ‘Yes’ were classified as current cigarette users; those who responded ‘No, but I was a smoker in the past’ were classified as former users; and those who answered ‘No, I have never smoked’ were classified as never users. An identical approach was used to classify hookah smoking status, assessed with the survey question, ‘Do you smoke hookah?’. Perceptions of hookah’s harm were categorized as more harmful than cigarettes, equally harmful as cigarettes, or less harmful than cigarettes; these were later collapsed into 2 categories: as or more harmful than cigarettes, and less harmful than cigarettes.

### Analyses

There were 2 primary outcomes of interest: hookah use and cigarette use. Multinomial regression models were constructed in order to determine the associations between each outcome and the independent variables. Relative risks (RRs) were reported from the multinomial regression models to facilitate interpretability of associations between predictors and smoking status categories. All models used the same demographic covariates of age, sex, length of stay in the country as a proxy for acculturation, alcohol use, health insurance, education, country of birth, marital status, and hookah health perception. An interaction variable between female sex and length of stay in the U.S. was created to assess whether the association between acculturation and cigarette use differed by gender.

Independent variables were selected a priori based on established sociodemographic determinants commonly used in tobacco research, as well as variables previously included in analyses using this dataset.^30^ Additional factors with well-documented associations with tobacco use, such as alcohol use and tobacco-related beliefs, were also incorporated based on the existing literature.^14,16^ Of the 919 survey respondents, only participants with complete responses for all variables included in each regression model were eligible for analysis. This resulted in analytic sample sizes of 458 for the cigarette use model and 443 for the hookah use model.

In both models, the reference category for the current user outcome was individuals who have never used the substance, while the reference category for the former user outcome was individuals who currently use the substance. Linearity of continuous variables was assessed by histogram and boxplot. Only participants without missing data were included in each model. For all statistical analysis, a *P* value of less than 0.05 was considered significant. All analyses were performed using R software version 4.4.0. Nagelkerke pseudo-R^2 values were 0.831 for the cigarette model and 0.933 for the hookah model; these higher values likely reflect the reduced effective sample sizes (N = 458 and N = 443, respectively), because the calculation is based only on complete cases with no missing values in the outcome or predictors.

## Results

A summary of participant demographics is shown in Table 1. The survey included 919 participants, averaging 39.7 years in age, with 57.8% (n = 526) identifying as female. In terms of immigration status, 76.4% (n = 697) were born in Syria and had spent an average of 17.7 years in the U.S.

**Table 1.** Participant characteristics, Syrian Americans, Southern California and Jacksonville, 2018-2019 (N = 919)

Characteristics	Frequency (%)^a^	Mean (SD)	[Min., Max.]
Age		39.7 (16.3)	[18.0, 85.0]
Sex			
Male	393 (43%)		
Female	526 (57%)		
Marital status			
Married	580 (64%)		
Single	272 (30%)		
Divorced/Widow/Other^b^	56 (6.1%)		
Education completed			
High school or less	430 (47.6%)		
Some college	154 (17%)		
2 year college degree	102 (11%)		
4 year college degree	148 (16%)		
Graduate or professional school	67 (7.4%)		
Birth history			
Born in Syria	697 (75.8%)		
Born in the United States	186 (20.2%)		
Born in another country	36 (3.9%)		
Length of stay in the United States^c^		17.7 (11.6)	[0.1, 53.4]
Language preference			
English	337 (37%)		
Arabic	558 (62%)		
Other	4 (0.4%)		
Has health insurance			
Yes	407 (45%)		
No	503 (55%)		
Alcohol use			
Daily or almost daily	11 (2.9%)		
Once or twice a week	75 (20%)		
3 or 4 times a week	33 (8.6%)		
Once or twice a month	141 (37%)		
Less than once a month	122 (32%)		
Cigarette use			
Current user	141 (16%)		
Years smoking		21.7 (13.0)	[1.0, 55.0]
Age when started smoking		22.4 (9.2)	[10.0, 67.0]
Number of cigarettes smoked daily		15.3 (10.0)	[1.0, 50.0]
Former user	102 (11%)		
Age when started smoking		19.9 (5.6)	[1.0, 46.0]
Age when stopped smoking		37.9 (11.4)	[15.0, 65.0]
Number of cigarettes smoked daily		17.0 (15.7)	[1.0, 80.0]
Never user	645 (73%)		
Hookah use			
Current user	323 (37%)		
Age when started smoking		26.1 (10.8)	[10.0, 61.0]
Former user	112 (13%)		
Never user	445 (51%)		
Dual use			
Current dual user (hookah + cigarettes)	27 (2.9%)		
Current single user (hookah or cigs only)^d^	316 (34.4%)		
Former user (hookah and/or cigs)^e^	71 (7.6%)		
Never used either	314 (34.2%)		
Missing/unknown	191 (20.8%)		

^a^Percentage is calculated using total number of participants who completed field.

^b^Includes divorced (2%), widowed (3.9%), and other responses (0.3%).

^c^Calculated using date immigrated to the United States and date of survey completion.

^d^Includes 235 current hookah-only users and 81 current cigarette-only users.

^e^Includes 28 former dual users and those who quit one product but not the other.

Table 1 also summarizes cigarette and hookah use. Beginning with cigarette use, 16% (n = 141) were current users, 11% (n = 102) were former users, and 73% (n = 645) never smoked cigarettes. Current users started smoking at an average age of 22.4 years, while former users began earlier, at average age 19.9, and quit at an average age of 37.9. Regarding hookah, 37% (n = 323) reported current use, 13% (n = 112) former use, and 51% (n = 445) as having never used. As Table 2 summarizes, among current hookah users, 19% reported daily use, with an additional 29% using 3-5 days per week. Most (77%) began using hookah as adults, though nearly a quarter initiated use before age 18. The top reasons for hookah use were social occasions/custom (79%, n = 251), followed by enjoyment of flavors (39%, n = 133). The majority of the sample (77%, n = 240) reported perceiving hookah to be as or more harmful than cigarettes.

**Table 2.** Characteristics among current hookah users, Syrian Americans, Southern California and Jacksonville, 2018-2019 (N = 919)

Characteristics	Frequency (%)
Use frequency– days per week	
Once	69 (22%)
Twice	37 (12%)
3 days	53 (17%)
4 days	32 (10%)
5 days	44 (14%)
6 days	16 (5.2%)
Daily	57 (19%)
Reasons for hookah use	
Social occasions/Custom	251 (79%)
Likes the flavor	131 (41%)
“I cannot stop”	31 (9.7%)
Healthier than cigarettes	7 (2.2%)
Other (unspecified)	13 (4.1%)
Hookah start age	
Adult (18+ years)	237 (77%)
Adolescent (<18 years)	69 (23%)
Perceptions about hookah harm	
Hookah is as or more harmful than cigarettes	240 (77%)
Hookah is less harmful than cigarettes	70 (23%)

Multinomial regression analysis for the primary outcomes regarding current (Column 1) and former (Column 2) cigarette use among the sample is shown in Table 3. In the model comparing current cigarette users to never users (Table 3, Column 1), older individuals had a higher risk of cigarette use compared to younger individuals (RR = 1.03 for 1-year increase, *P* = 0.023). Women were significantly less likely to use cigarettes compared to men (RR = 0.09, *P* < 0.001), and a longer length of stay in the U.S. was modestly protective against cigarette use overall (RR = 0.95, *P* = 0.003). However, a significant interaction between female sex and length of stay (RR = 1.06, *P* = 0.018) suggests that this protective effect of acculturation was weaker—or even reversed—among women, for whom longer U.S. residency was associated with a higher likelihood of smoking. Individuals who consumed alcohol were significantly more likely to be current cigarette users (RR = 2.87, *P* < 0.001). Additionally, individuals who believed that hookah is less harmful than cigarettes were at a lower risk of cigarette use (RR = 0.21, *P* = 0.016).

**Table 3.** Multinomial logistic regression of cigarette use predictors, Syrian Americans, Southern California and Jacksonville, 2018-2019 (N = 458)

Characteristic	Current cigarette user (ref: never user)		Former cigarette user (ref: current user)	
	RR [95% CI]	*P*-value	RR [95% CI]	*P*-value
Age, 1-year increase	1.03 [1.00-1.05]	**0.023**	1.02 [0.99-1.05]	0.12
Sex, female	0.09 [0.03-0.25]	**<0.001**	1.28 [0.28-5.74]	0.75
Married	0.85 [0.44-1.64]	0.62	0.76 [0.33-1.80]	0.54
Completed at least some college	0.60 [0.35-1.04]	0.068	2.07 [1.02-4.22]	**0.045**
Has health insurance	1.11 [0.62-1.98]	0.73	0.99 [0.46-2.13]	0.98
Drinks alcohol	2.87 [1.61-5.14]	**<0.001**	0.65 [0.30-1.41]	0.28
Believes hookah is less harmful than cigarettes	0.21 [0.06-0.75]	**0.016**	5.58 [1.36-22.8]	**0.017**
Country of birth, Syria	1.96 [0.38-10.2]	0.42	0.57 [0.07-4.52]	0.59
Years lived in the U.S. (LOS)	0.95 [0.92-0.98]	**0.003**	1.03 [0.99-1.07]	0.12
Female*LOS	1.06 [1.01-1.11]	**0.018**	0.97 [0.90-1.03]	0.31

The reference category was ‘Never user’ for the ‘Current cigarette user’ outcome (Column 1). The reference category was ‘Current cigarette user’ for the ‘Former cigarette user’ outcome (Column 2). Bolded values indicate statistical significance (p≤0.05).

In the model comparing former cigarette users to current users (Table 3, Column 2), individuals who completed at least some college were twice as likely to have quit cigarette use, based on their former user status (RR = 2.07, *P* = 0.045). Believing that hookah is less harmful than cigarettes was also associated with a greater likelihood of having quit cigarette use (RR = 5.58, *P* = 0.017), as indicated by former user status.

A multinomial model summarizing factors predicting current (Column 1) and former (Column 2) hookah use can found in Table 4. In the model comparing current hookah users to never users (Table 4, Column 1), older individuals were less likely to use hookah than younger individuals (RR = 0.92, *P* < 0.001). Those who drink alcohol were approximately twice as likely to use hookah compared to those who do not (RR = 2.16, *P* = 0.010). Having health insurance was associated with a lower likelihood of hookah use (RR = 0.51, *P* = 0.016), as was having attended at least some college (RR = 0.60, *P* = 0.047). Conversely, married individuals were more likely to use hookah (RR = 3.40, *P* = 0.001). Additionally, individuals who believed that hookah is less harmful than cigarettes were nearly ten times more likely to use hookah (RR = 9.98, *P* < 0.001). Current cigarette users were less likely to use hookah (RR = 0.41, *P* = 0.014), whereas former cigarette users were more likely to use hookah (RR = 2.56, *P* = 0.033).

**Table 4.** Multinomial logistic regression of hookah use predictors, Syrian Americans, Southern California and Jacksonville, 2018-2019 (N = 443)

Characteristic	Current hookah user (ref: never user)		Former hookah user (ref: current user)	
	RR [95% CI]	*P*-value	RR [95% CI]	*P*-value
Age, 1-year increase	0.92 [0.90-0.95]	**<0.001**	1.00 [0.96-1.04]	0.94
Sex, female	1.41 [0.50-4.01]	0.52	0.53 [0.13-2.22]	0.38
Married	3.40 [1.61-7.17]	**0.001**	0.37 [0.13-1.01]	0.053
Completed at least some college	0.60 [0.36-0.99]	**0.047**	1.75 [0.84-3.64]	0.14
Has health insurance	0.51 [0.30-0.88]	**0.016**	2.56 [1.14-5.68]	**0.021**
Drinks alcohol	2.16 [1.21-3.86]	**0.010**	1.35 [0.62-2.96]	0.46
Believes hookah is less harmful than cigarettes	9.98 [4.08-24.5]	**<0.001**	0.11 [0.02-0.49]	**0.004**
Country of birth, Syria	1.91 [0.48-7.63]	0.36	2.30 [0.23-23.0]	0.48
Years lived in the U.S. (LOS)	0.99 [0.95-1.02]	0.49	1.01 [0.96-1.07]	0.61
Female*LOS	1.00 [0.96-1.05]	0.94	1.00 [0.94-1.08]	0.92
Cigarette use				
Current cigarette user	0.41 [0.20-0.84]	**0.014**	—	—
Former cigarette user	2.56 [1.08-6.09]	**0.033**	1.32 [0.43-4.08]	0.63

The reference category was ‘Never user’ for the ‘Current hookah user’ model (Column 1). The reference category was ‘Current user’ for both the outcome (Former hookah user) and the cigarette use predictor (Column 2).

In the model comparing former hookah users to current users (Table 4, Column 2), having health insurance and believing that hookah is less harmful than cigarettes were both significantly associated with former user status. Having health insurance was associated with a greater likelihood of having quit hookah, as suggested by former user status (RR = 2.56, *P* = 0.021), while believing that hookah is less harmful than cigarettes was associated with a lower likelihood of hookah cessation (RR = 0.11, *P* = 0.004).

## Discussion

This is the first study to investigate tobacco use among Syrian Americans in the U.S., a growing population with a high prevalence of tobacco use. In contrast to earlier studies on Arab Americans, this research provides an in-depth analysis of disaggregated data with a comparatively large sample size. The findings provide valuable insight on tobacco use within this immigrant population and has important implications for tobacco use interventions.

### Prevalence of Cigarette and Hookah Use

Within this sample, 16% of participants were current cigarette users and 37% of participants were current hookah users. The prevalence of current cigarette and hookah use among Syrians in 2019 was 16.4% and 29.3%, respectively.^31^ The CDC reported that in 2018, the year most participants responded to the study survey, the prevalence of current cigarette use among adults in the U.S. was 13.9%.^32^ The prevalence of current smoking pipe users, which includes hookah, water pipe, and regular pipes, among adults in the U.S. was 0.9% in 2021.^33^ One study found an 18% prevalence of cigarette smoking among foreign-born Arab Americans using data from the 2000-2014 National Health Interview Survey.^10^ While there is limited research on rates of hookah use among Arab American adults, one study from Colorado found a prevalence of 21%.^34^ Overall, this study’s sample had a higher prevalence of cigarette use compared to U.S. adults, but had very similar rates of cigarette use compared to Arab Americans and Syrians. In terms of hookah use, this sample of Syrian Americans showed higher rates compared to the U.S. population, Arab Americans, and Syrians. Only 11% of Syrian Americans reported former cigarette use, and 13% reported former hookah use. The lack of data on hookah smoking cessation in other studies highlights the need for further research. Syrian Americans may be at higher risk of adverse health effects associated with tobacco use, underscoring the importance of targeted tobacco prevention and cessation programs for this population.

### Predictors of Tobacco Cessation

Participants who completed at least some college were more likely to be a former cigarette user as compared to a current user. This result aligns with the well-documented trend in the U.S. that individuals with higher levels of educational attainment are more likely to quit smoking.^35,36^ Additionally, those who believe that hookah use is less harmful than cigarette use were at higher odds of being former cigarette users. While the relationship between cigarette and hookah use remains nuanced, one study suggested that hookah may serve as a substitute for cigarette use in the initiation phase of cigarette cessation among young people.^37^ This may explain the association found in this study: participants attempting to quit cigarettes may use hookah as a cessation aid.

### Dual Use

Many studies have suggested that cigarette use is a predictor of hookah use, or of being a dual user of both cigarettes and hookah.^11,14-16,18,21,38^ In contrast, this analysis showed that current cigarette users had a lower risk of being a concurrent hookah user, when compared to those who never use. These results may suggest that Syrian Americans use cigarettes and hookah differently, so interventions should address each type of tobacco use separately.

The analysis also showed that former cigarette users had a higher risk of being a current hookah user. The preexisting literature has demonstrated both possible chronologies: that hookah use may predict later cigarette use, and vice versa. This study supports the hypothesis that cigarette use may be the precursor to alternative tobacco use, including hookah smoking.^16,19,22,39,40^ Other studies have suggested that hookah use may increase cessation barriers and nicotine dependence among concurrent cigarette users.^20^ This study’s results indicate that former cigarette users may choose to continue hookah use for the benefits of participating in the social pastime, and not necessarily due to nicotine dependence.

### Hookah Perception

Believing hookah is less harmful than cigarettes was the strongest predictor of current use, with believers nearly 10 times more likely to smoke than never users. This effect was even stronger than reported in prior U.S. studies linking harm perception to hookah use.^14,16,19,41^ Only 2.2% of participants cited “healthier than cigarettes” as a reason for hookah use, and 77% perceived it as equally or more harmful. This aligns with other studies suggesting that misperceptions of harm may not be the main barrier to cessation.^21,38^ Since 79% reported smoking for social reasons, the perceived social benefits may outweigh health concerns.^13,22^ Still, the strong association (RR = 9.98) suggests that knowledge gaps remain influential. Accurately perceiving hookah’s risks has been linked to lower use and greater quit intentions among Arab Americans and may support cessation among Syrian Americans.^26^

### Predictors of Hookah Cessation

Having health insurance was positively associated with former hookah use as opposed to current use. Since insured participants have better healthcare access, clinical encounters may be key opportunities for smoking-cessation education and intervention.^30^ Furthermore, incorrectly believing that hookah is less harmful than cigarettes was negatively associated with former hookah use as compared to current hookah use. This suggests that while underestimating harm may not drive use, understanding its health risks could support cessation.

### Sex and Acculturation

Each additional year foreign-born participants lived in the U.S. was associated with a lower risk of current cigarette use compared to never users. This supports other studies on Arab Americans where a longer length of stay in the U.S. is associated with an increase in attempts to quit cigarette smoking.^8,10^ The results affirm the literature demonstrating a positive relationship between acculturation in the U.S. and smoking cessation among immigrants from countries with high tobacco use.^42^

Males were at a significantly higher risk of being current cigarette users, but sex was not significantly associated with hookah use. This finding is expected given the disproportionately higher rate of smoking found among Syrian and Arab American males when compared to their female counterparts.^5-7,10^ In Syria, cigarette smoking among women may be perceived much more negatively than among men.^5,43^ These social norms may persist among Syrian Americans even after relocating to the U.S. and may explain the disproportionate prevalence by sex.^10^

These cultural norms may also explain the significant interaction found in our study, where sex influenced the effect of length of stay on cigarette use, with females at higher risk. Syrian American women who have spent more years in the U.S. may be more acculturated to a culture where female smoking is viewed more favorably than in their country of origin.^44^ The sample revealed significant trends in female cigarette use, but not in female hookah use. In accordance with other authors, this may be due to the relatively more favorable perception of female hookah use in Arab culture.^5,43^

### Age

The average smoking initiation ages for current cigarette users, former users, and hookah users were 22.4, 19.9, and 26.1 years, respectively. In contrast, the U.S. average is 15.3 years,^1^ while a 2006 Syrian study found initiation at 17.9 (men) and 22.5 (women) for cigarettes, and 22.5 (men) and 28.9 (women) for hookah.^7^ The higher initiation ages in this study may reflect the absence of participants under 18.

Older individuals had a higher risk of cigarette use and a lower risk of hookah use than younger adults. This mirrors CDC data showing tobacco use rises with age until 65.^45^ Older U.S. adults are also less likely to attempt quitting or access cessation resources.^46-48^ These factors may explain the link between older age and smoking in this sample.

Most (77%) participants began using hookah as adults, though nearly a quarter initiated use before age 18 (Table 3). While no studies have assessed which age groups are most likely to use hookah, preliminary studies have demonstrated a high prevalence of hookah smoking among college students in the U.S. and Syria.^28,38,49^ In the U.S., this trend is likely driven by the concentration of hookah establishments near college campuses, targeted online marketing, and the popularity of hookah lounges among young adults aged 18-21.^14,50,51^ These social and environmental factors may lose relevance with age, potentially explaining the negative association between age and hookah use observed in this study.

Additionally, older participants were more likely to be former cigarette users but less likely to be former hookah users. The average quitting age (37.9 years) mirrored the U.S. average of 38 years,^52^ suggesting similar age-related smoking patterns in the sample.

### Education and Hookah Use

Completing at least some college was associated with lower odds of current hookah use among Syrian Americans. In contrast, U.S. studies report higher hookah use among adults with some college education,^16,41^ likely due to the concentration of hookah lounges near campuses—often cited as common initiation sites.^50,53^ However, Kassem et al found Arab Americans typically first try hookah at home or a friend’s house, influenced by friends and family.^40^ Studies also show Arab American youth have higher hookah use rates than non-Arab peers.^38,49,54^ This cultural emphasis may explain the inverse association between college attendance and use in this group. While many Americans first encounter hookah in college, Syrian Americans often do so through family and social traditions—a pattern reflected in our finding that 79% cited “social occasions/custom” as their reason for use. Culturally competent, community-based cessation programs are needed to address these strong social influences.

### Alcohol Use

Sample participants who drink alcohol were at a higher risk of reporting both current cigarette use and current hookah use. The significant association between the use of tobacco products and alcohol consumption within the U.S. is well known.^55-61^ This finding contributes to the robust literature on this relationship and highlights the importance of addressing alcohol use as a part of both hookah and cigarette cessation.

### Marital Status

Being married was a significant predictor of hookah use among Syrian Americans but was not significant for cigarette use. Previous studies found that married individuals had more positive attitudes toward hookah but not cigarettes.^28,43^ Another study suggested that compared to married Syrian women, unmarried Syrian women are more likely to cite traditions and norms as a reason that they do not smoke cigarettes.^62^ This suggests married Syrian women’s smoking behavior may be shaped more by marital expectations than broader social norms. Alternatively, unmarried Syrian women may experience greater social scrutiny in public spaces, making them more vulnerable to societal expectations that discourage women smoking. This pattern contradicts studies on smoking status in the U.S., which suggest individuals who are married are less likely to smoke than any other marital status group.^33,63^ This indicates a predictive factor unique to Syrian Americans that should be understood and targeted by intervention programs.

### Limitations

This study has limitations typical of a survey-based methodology. The use of convenience sampling might restrict the ability to generalize findings to the broader Syrian American and Arab American populations. Despite the anonymity of the survey, the stigma surrounding tobacco use may have led to underreporting. Additionally, selection bias may be present, as the sample only includes individuals who chose to participate in the survey. Although data were collected in 2018-2019, there remains a scarcity of U.S.-based research focused specifically on tobacco use and cessation among Syrian Americans, and these findings provide important population-specific insight in the absence of more recent data. Despite these limitations, the study’s findings are critical to develop a better understanding of tobacco use among Syrian Americans.

## Conclusion

This study offers important insights into factors driving tobacco use among Syrian Americans to guide cessation efforts. Given the strong cultural ties to hookah use among Syrian Americans, culturally-competent, clinical- or community-based programs could be particularly beneficial in addressing hookah cessation. Health promotion campaigns and individualized clinical counseling based on the attitudes toward smoking identified in this study could significantly enhance the health of Syrian Americans. Additional research is needed to further address the high prevalence of tobacco use in this growing population in the U.S.

## Supplemental Material

Supplemental Material - Predictors of Tobacco Use Behaviors Among Syrian Americans Supplemental Material for Predictors of Tobacco Use Behaviors Among Syrian Americans by Isabel Nakoud, Cyrene Arputhasamy, Catherine M. Crespi, Jovana F. Mahho, Mellissa Withers, Burt Cowgill in Tobacco Use Insights

## Data Availability

The data underlying this study are available from the corresponding author upon reasonable request.*
