# Supplementary material for: Predictors of Tobacco Use Behaviors Among Syrian Americans
Source: Tob Use Insights. 2026 Jun 3;19:1179173X261423430. doi: 10.1177/1179173X261423430 (PMC13234375; doi:10.1177/1179173X261423430)
Supplement: Supplemental Material - Predictors of Tobacco Use Behaviors Among Syrian Americans [file sj-pdf-1-tui-10.1177_1179173X261423430.pdf]

Chronic disease in Syrian Americans

Please complete the survey below.

Thank you!

We invite you to participate in a research study conducted by Jamil Samaan from the Keck School of Medicine of USC, in Los Angeles, California. We hope to learn about the prevalence of chronic diseases in Syrian-Americans. The results of this survey will be used to develop future studies to better meet the health care needs of Syrian-Americans.
If you decide to participate, you are asked to complete this survey which will take about 15 minutes of your time. You may choose to skip or stop answering any question that you want to. This survey is anonymous and no one will be able to link your answers back to you. Please do not include your name or other information that could be used to identify you in the survey responses.Your participation is completely voluntary. There are no consequences to you if you decline to participate. You will not be paid for participating in the study. If you have any questions about the research or the survey, you may contact a member of our research team:

Lead researcher: Jamil Samaan 714-580-9679 or jsamaan@usc.edu
Principal Investigator: Melissa Withers Ph.D. (323) 865-0127 or mwithers@usc.edu

If you have questions or concerns about your rights as a research participant, please call the Health Sciences Institutional Review Board at (323) 223-22340 or email at irb@usc.edu.

Have you participated in this survey before? ◯ Yes ◯ No

If you have participated in this survey before, please stop filling out this survey

Age (Years) ______________________________

If under 18 years old, please stop filling out this survey

Are both of your parents of Syrian decent? ◯ Yes ◯ No

If both of your parents are not of Syrian decent, please stop filling out this survey

Today's Date (Month/Day/Year) __________________

Sex
- ○ Male
- ○ Female

Marital status
- ○ Married
- ○ Single
- ○ Divorced/Separated
- ○ Widow
- ○ Other

Height (cm or Feet and Inches) __________________ (If box is red, please disregard and continue)

Weight (Kg or Pounds) __________________ (If box is red, please disregard and continue)

Religion
- ○ Christian
- ○ Muslim
- ○ Other
- ○ Prefer not to answer

What city do you currently live in? __________________ (If box is red, please disregard and continue)

Do you have children?
- ○ Yes
- ○ No

If yes, how many children do you have?

○ 1 ○ 2 ○ 3 ○ 4 ○ 5 ○ 6 ○ 7 ○ 8 ○ 9 ○ 10 ○ 11 ○ 12 ○ 13

What state do you currently live in? __________________ (If box is red, please disregard and continue)

Were you born in Syria?
- ○ Yes
- ○ No

If no, what country were you born in? __________________ (If box is red, please disregard and continue)

If you were born in Syria, what city/town are you from? __________________

If you were not born in the United States, what month and year did you immigrate to the United States? __________________ (If box is red, please disregard and continue)

What language do you most often use at home?
- ○ English
- ○ Arabic
- ○ Other

What language do you prefer using?
- ○ English
- ○ Arabic
- ○ Other

Do you prefer healthcare providers who speak Arabic?
- ○ Yes
- ○ No preference
- ○ No

What do you do for a living? __________________ (If box is red, please disregard and continue)

What is the highest level of education that you have completed?
- ○ Primary School (1st-6th)
- ○ Secondary School (7th-9th)
- ○ High School (10th-12th)
- ○ Some college
- ○ 2 year college degree
- ○ 4 year college degree
- ○ Graduate or Professional School

Do you have health insurance?
- ○ Yes
- ○ No
- ○ Do not know/Do not remember

If you have health insurance, what kind? (Example: Medical, Medicare, Kaiser Permanente, Blue Cross, etc.) __________________ (If box is red, please disregard and continue)

If you do not have health insurance, what are the reasons? (Check all that apply)
- ☐ I cannot afford it
- ☐ I do not know how to apply for health insurance
- ☐ I do not get sick enough to need insurance
- ☐ Other reasons

If you have other reasons, please list them. __________________

Do you drink alcohol?
- ○ Yes
- ○ No

About how often do you drink an alcohol beverage?
- ○ Daily or almost everyday
- ○ Once or twice a week
- ○ Three or four times a week
- ○ Once or twice a month
- ○ Less often than once a month

Do you currently smoke cigarettes?
- ○ Yes
- ○ No, but I was a smoker in the past
- ○ No, I have never smoked

How long have you been smoking cigarettes? __________________

At what age (in years) did you start smoking cigarettes? __________________

How many cigarettes a day do you smoke on average? __________________

At what age did you start smoking cigarettes ? __________________

At what age did you stop smoking cigarettes? __________________

How many cigarettes a day did you used to smoke on average? __________________

Do you believe smoking hookah is as harmful, more harmful or less harmful to your health compared to smoking cigarettes?
- ○ Same
- ○ More
- ○ Less

Do you smoke hookah?
- ○ Yes
- ○ No, but I smoked hookah in the past
- ○ No, I have never smoked hookah

How long have you been smoking hookah? __________________

At what age did you start smoking hookah? __________________

How many days a week do you smoke hookah, on average?
- ○ 1
- ○ 2
- ○ 3
- ○ 4
- ○ 5
- ○ 6
- ○ 7

Why do you smoke hookah? (Mark all that apply)
- ☐ I like the flavor
- ☐ Cultural Reasons
- ☐ Social Occasions
- ☐ Healthier than cigarettes
- ☐ I cannot stop
- ☐ Other reasons

If you marked other reasons, please list your reasons ______________________________

Has a doctor ever told you that you have any of the following conditions?

Diabetes	○ Yes	○ No	○ Prefer not to answer
High blood pressure	○ Yes	○ No	○ Prefer not to answer
High cholesterol	○ Yes	○ No	○ Prefer not to answer
Cancer	○ Yes	○ No	○ Prefer not to answer
Lung disease	○ Yes	○ No	○ Prefer not to answer
Kidney disease	○ Yes	○ No	○ Prefer not to answer
Arthritis	○ Yes	○ No	○ Prefer not to answer
Migraines	○ Yes	○ No	○ Prefer not to answer
Heart disease	○ Yes	○ No	○ Prefer not to answer
Stroke	○ Yes	○ No	○ Prefer not to answer
Liver disease	○ Yes	○ No	○ Prefer not to answer
Thyroid disease	○ Yes	○ No	○ Prefer not to answer
Heartburn	○ Yes	○ No	○ Prefer not to answer
Herniated disc	○ Yes	○ No	○ Prefer not to answer
Gout	○ Yes	○ No	○ Prefer not to answer

Please list conditions not shown above. ______________________________

If you have had cancer, what type?
- ☐ Colon
- ☐ Breast
- ☐ Prostate
- ☐ Lung
- ☐ Blood
- ☐ Liver
- ☐ Kidney
- ☐ Skin
- ☐ Bladder
- ☐ Other

Please list any cancers not shown above. ______________________________

At what age were you diagnosed with high blood pressure? ______________________

At what age were you diagnosed with high cholesterol? ______________________

At what age were you diagnosed with diabetes? ______________________

Diet modification is essential for the management of diabetes
- ○ Strongly agree
- ○ Agree
- ○ Neutral
- ○ Disagree
- ○ Strongly disagree

Regular exercise can be effective for the management of diabetes
- ○ Strongly agree
- ○ Agree
- ○ Neutral
- ○ Disagree
- ○ Strongly disagree

Have you experienced any of the following in the past year? (Check all that apply)
- ☐ Foot numbness
- ☐ Foot tingling
- ☐ Cold/Hot sensations in your feet
- ☐ Changes/loss in vision
- ☐ Foot/Leg amputation(s)

Do you take any medications for your heartburn?
- ○ Yes
- ○ No

If yes, what medications do you take for your heartburn?
- ☐ Prilosec OTC (Omeprazole)
- ☐ Nexium (Esomeprazole)
- ☐ Prevacid (Lansoprazole)
- ☐ Protonix (Pantoprazole)
- ☐ Aciphex (Rabeprazole)
- ☐ Pepcid
- ☐ Zantac
- ☐ Tums
- ☐ Other

If other, what medication(s) do you take for your heartburn? ______________________

How long have you been taking these medications for your heartburn?
- ○ Less than 1 week
- ○ 1-2 weeks
- ○ 2 weeks-1 month
- ○ 1 month-6 months
- ○ 6 months-1 year
- ○ More than 1 year

Is there a history of cancer in your family? (father, mother, brother, sister, grandparents)	◯ Yes<br>◯ No<br>◯ Do not know/Do not remember
If there is a family history of cancer, what type?	☐ Colon<br>☐ Breast<br>☐ Prostate<br>☐ Lung<br>☐ Blood<br>☐ Liver<br>☐ Kidney<br>☐ Skin<br>☐ Bladder<br>☐ Other
Have you been seen by a healthcare provider for a routine medical examination in the past year?	◯ Yes<br>◯ No
If you have not had a routine medical examination in the past year, what are the reasons? (Check all that apply)	☐ I do not feel sick<br>☐ I can not afford it<br>☐ I do not see the need for yearly check ups<br>☐ I am too busy<br>☐ I have trouble understanding my doctor<br>☐ Other
Have you ever had a blood test for diabetes or cholesterol?	◯ Yes<br>◯ No<br>◯ Do not know/Do not remember
If yes, what month and year was your most recent blood test?	__________________________
Have you ever undergone a colonoscopy (Colon Cancer screening procedure) ?	◯ Yes<br>◯ No<br>◯ Do not know/Do not remember
If yes, what month and year was your most recent colonoscopy (Colon Cancer screening procedure)?	__________________________
Have you ever undergone a mammogram (breast cancer scan)?	◯ Yes<br>◯ No<br>◯ Do not know/Do not remember
If yes, what month and year was your most recent mammogram (breast cancer scan)?	__________________________
What medications are you currently taking?	☐ Atorvostatin<br>☐ Simvastatin<br>☐ Metformin<br>☐ Hydrocodone/Acitaminophen<br>☐ Metoprolol<br>☐ Losartan<br>☐ Hydrochlorothiazide<br>☐ gabapentin<br>☐ levothyroxine<br>☐ Lisinopril<br>☐ Omeprezole<br>☐ Amlodipine<br>☐ Levothyroxine<br>☐ Metoprolol<br>☐ Advil/Alieve<br>☐ Meloxicam

Please list any medications not listed above	__________________________
